# Cancer Prevention Strategies for Nepal

**DOI:** 10.31729/jnma.7014

**Published:** 2022-01-31

**Authors:** Deepak Sundar Shrestha, Richard R. Love, Bishnu Dutta Paudel

**Affiliations:** 1 Richa Bajimaya Memorial Foundation, Kathmandu, Nepal; 2 Department of Computer Science, Marquette University, Milwaukee, Wisconsin, U.S.A.; 3 Bhaktapur Cancer Hospital, Bhaktapur, Nepal

**Keywords:** *alcohol drinking*, *betel nut*, *helicobacter pylori*, *human papilloma virus*, *tobacco smoking*

## Abstract

In Nepal, the commonest major malignancies and causes of cancer death are lung, cervix, stomach, breast, head and neck (lip, mouth, pharynx, larynx), gallbladder, ovary and liver. There are seven cancer-causative exposures which should be the focus of attention such as; tobacco smoking in 29% of men, and 6% of women, solid fuel burning in 69% of homes (multiple cancers), betel-nut chewing in 40 % of men and 3% of women (head and neck cancers), alcohol abuse (liver and other cancers), Human Papilloma Virus (cervical cancer), Helicobacter pylori (stomach cancer) and Hepatitis B virus (liver cancer). To better address these reducible exposures, we suggest greater targeted strategies in three areas: Public health messaging for tobacco, solid-fuel burning, betel-nut, and alcohol; national policies for Hepatitis B virus vaccination; and analytic epidemiological and interventional research for Human Papilloma Virus and helicobacter.

## INTRODUCTION

The major goals for cancer prevention in Nepal are similar to those among high-income countries, but there are major cancer-causing exposure differences, and social contexts, which, of necessity, should direct country-specific approaches. The goal of cancer prevention is to decrease premature mortality from malignancies by interventions which decrease incidence rates in middle-aged and young populations. Such interventions are primarily directed to decreasing critical exposures.^1,2^ This communication will first consider the useful descriptive and analytic epidemiology of cancer frequencies in Nepal and known causes and then propose targeted and prioritized messaging, public policy, and research approaches.

## SELECTED RELEVANT DESCRIPTIVE AND ANALYTIC EPIDEMIOLOGY FOR NEPAL

The age pyramid for Nepal is striking for demonstrating the young age of the population, which circumstances have huge implications for allocations of efforts and resources in cancer prevention.^3^ Sixty and one-half percent of the population in 2019 was under age 30 29.3% men and 30.2% women.

The descriptive epidemiology for major cancer case burdens and deaths is presented (Table 1 and 2.)

**Table 1:** Major cancer cases projected for Nepal 2020.^4^

Site	n (%)
Lung	2,505 (12.2)
Cervix	2,244 (10.9)
Breast	1,973 (9.3)
Stomach	1,552 (7.6)
Head and neck	1,274 (6.2)
Gallbladder	1,015 (4.9)
Ovary	594 (2.9)
Liver	524 (2.6)

**Table 2:** Nepal, percentages of all cancer deaths from selected cancers.^4^

Site	n (%)
Lung	17.0
Cervix	11.0
Stomach	10.2
Breast	7.7
Gallbladder	5.5
Head and neck	5.1
Liver	3.7
Ovary	2.8

What is particularly striking are gender differences: incidence rates of stomach cancer are two-fold higher in men, those of gallbladder are two-fold higher in women, and those of liver are four-fold higher in men. ^4^

Of these major cancers listed in tables 1 and 2, while the role of tobacco smoking in lung and head and neck cancers is well-known, the documented contributions of this exposure to increased risks for cancers of the stomach, cervix, breast, ovary, and gallbladder are less highlighted and discussed.^5-10^

The trends are for decreasing smoking in the last decade (7-11%), but markedly so for women, so part of the lung and other cancer story in women can be from previously much higher rates of smoking.^11,12^ The smoking problem is compounded by the preponderance of data indicating that passive smoking—exposure to second-hand smoke—is associated with increased risk of lung and breast cancers. ^13^

While for acute and chronic respiratory health reasons, there has been increased attention to the problem of solid fuel burning in 69% of Nepalese homes, the likely contribution of this home air pollution to lung cancer in women in particular has not been emphasized.^14^ Further compounding interpretation of the contributions of previous personal tobacco smoking, passive smoking, and home solid fuel-burning to lung cancer rates, is the uncertain, but strongly suspected contribution of general urban air pollution, particularly in the Kathmandu valley.^15^

Betel-nut chewing in various forms (particularly with tobacco as a PAAN) is associated with head and neck cancers of the lip, mouth, and pharynx.^16^ The prevalence of use rises from 23% in men at age 20 to almost half of men in Nepal at age 50; in women the prevalence of use rises to 14% at age 50.^17^ Easy access and affordability currently, make this a serious problem.

Alcohol contributes significantly to the development of cancers of the liver, head and neck, esophagus, colon, and breast.^18^ In Nepal, recent data report that 38.6% of men, and 10.8% of women are current drinkers and 12% of men drink daily or almost daily, and engage in heavy episodic drinking.^19^

Human papilloma virus is clearly causative of cervical and head and neck cancers.^20,21^ A central role of Helicobacter pylori bacterial infection in stomach cancer is well described.^22^ Finally, the role of HBV infection in liver cancer is major.^23^

## PRIORITIZED PUBLIC HEALTH MESSAGING

Maintaining good health is overwhelmingly based on what individuals do for themselves. The foregoing epidemiology summary makes very clear that the major cancer burdens and their causes in Nepal are unquestionably addressable by better targeted public health activities in messaging and public policy. The public can benefit from more and creative messaging in four key areas: tobacco, betel-nut chewing, alcohol and solid fuel burning in homes.

## TOBACCO

Graphic warning labels on tobacco products have been consistently demonstrated to be effective in promoting quitting smoking, and in reducing initiation, especially with younger people, which situation is very relevant in Nepal (vide supra).^24-26^ Internationally, we have learned again some key lessons about public health messaging from the Covid pandemic: messages need to be consistent, repeated, and sensitive to equity issues. There are some areas for anti-tobacco messaging which have proven particularly effective (Table 3).

**Table 3:** Suggested strongest types of messaging about tobacco smoking.

Focus areas for effective anti-tobacco messaging Contents of cigarettes Addiction from cigarettes Suffering from cigarettes: Images of blackened lungs, neck with tumors, facial deformities with tumors, bones with holes from metastatic tumors Vignettes of patients suffering Social leaders speaking out against tobacco

## BETEL NUT CHEWING

This culturally grounded common habit is notably more prevalent in lower socioeconomic groups, and is increasing in younger Nepalis.^17,27,28^ The themes effective in anti-tobacco messaging would seem to be potentially useful in public health messaging about this habit. In particular, imaging lip, mouth, and pharynx tumors and their treatment consequences might be expected to resonate with younger Nepalis.

## ALCOHOL ABUSE

In Nepal, culturally, alcohol consumption has major roots in ethnic and religious traditions. These circumstances make potentially impactful messaging about alcohol a subject for careful research.

**Solid fuel-burning in homes**

There has simply not been enough public health attention and messaging about the pleomorphic adverse health consequences of solid fuel-burning which occurs in 69% of Nepali homes, and more frequently in rural communities.^14^ Women and children are disproportionately affected with pulmonary and cardiovascular chronic illnesses in addition to lung cancer from this exposure. Messaging themes focusing on children's health and gender equity may be notably effective.

## PUBLIC POLICY INITIATIVES

**Tobacco**

Four strategies have been significantly successful in reducing tobacco smoking globally:^24^

1. Increases in excise taxes on tobacco.
2. Required graphic warning labels on tobacco packaging.
3. Banning of tobacco industry marketing. And
4. Smoke-free laws that prohibit smoking in public and workplaces.

Nepal has made strong commitments to tobacco control in signing, ratifying and formally becoming a party to the WHO Framework Convention on Tobacco Control (FCTC). The tobacco product (Control and Regulatory) Act 2011 is the primary law governing tobacco control in the country. Continued commitment to tobacco control is evident in the framework convention on tobacco control strategy 2030 which specifies:

- Strengthening the legislation and policy environment
- Use of tax to finance development innovations
- Strict enforcement of legal provisions on tobacco control
- Protection of people from exposure to tobacco smoke
- A ban on sales to and by minors
- Increases in tobacco taxes
- Effective enforcement and implementation of impactful packaging
- A comprehensive ban of tobacco advertising, promotion and sponsorship
- Creation of a supportive environment for a tobacco-free generation
- A comprehensive system to provide tobacco cessation support to public
- Preventing interference of the tobacco industry in policy development
- Integrating tobacco control as a priority agenda in other health and non-health initiatives
- Introducing tobacco control into school and university curriculum.
- Engagement of civil society in enforcing tobacco control laws
- Strong media engagement for advocacy and enforcement of laws and regulations and enhance awareness on danger of tobacco use

What is critical now is active pursuit of this strategy and these specific activities. We believe that in particular, greater fines for breaking the laws on sales, empowerment of broad civil societal groups in tobacco control, increasing the age for legal purchase of tobacco to 21, development of better age verification tools, and prohibition of tobacco product use in all forms of media should be considered.

**Betel-Nut chewing**

While betel nut chewing has long been a culturally acceptable practice for religious purposes, the majority of users seem to be unaware of its harmful effects.^29^ Strategies similar to those which have been so successful with tobacco noted above, need to be explored.

**Alcohol**

The WHO considers public policies in 3 areas related to alcohol to be among 16 Best Buys for prevention and control of noncommunicable diseases: i. Excise taxes on alcoholic beverages; ii. Bans on alcohol advertising; and iii. Restrictions on sales of alcoholic beverages.^30^ Nepal needs additional laws addressing production and sales of locally brewed alcoholic beverages. Further educational efforts regarding the adverse health effects of alcohol are needed.

**Vaccination**

HBV: The estimated population Hepatitis B surface antigen seroprevalence is 2-4%.^31^ A national hepatitis B vaccination program among children was conducted in 2002-04, and in its evaluation, low rates of hepatitis B infections were found in children before and after immunization.^32^ The government of Nepal, with the assistance of GAVI introduced Hepatitis B vaccine from 2002 to 2004. It is currently administered as penta-valent vaccine along with Hemophilus influenza B (HiB) and Diphtheria Pertussis and Tetanus (DPT) vaccines at 6, 10 and 14 weeks of age. Currently there is no policy for antibody titre testing and booster dose administration.

HPV: Globally, vaccination against Human Papilloma Virus has been proven to be an effective means to prevent cervical cancer. As tables 1 and 2 above indicate, cervical cancer is a major malignancy and cause of cancer death among Nepali women. There have been successful trial HPV vaccination programs in a few districts of Nepal, and there have been strong recommendations to implement HPV vaccination as part of regular immunization program.^33,34^

## ANALYTIC EPIDEMIOLOGIC AND IMPLEMENTATIONAL RESEARCH

The two-fold higher rates of stomach cancer in men, and the two-fold higher rates of gallbladder cancer in women would seem to be appropriate questions to focus on in analytic epidemiologic studies.

Given the known major role for Helicobacter pylori in stomach cancer, investigation of population-based descriptive and analytic epidemiology and then pilot interventional studies addressing this infection are needed.^35^

**Funding**

Nepal is heavily dependent on international funding for health. Internationally, the epidemiologic transition to greater non-communicable disease (NCD) burdens in countries like Nepal is getting more attention, but NCDs are still way underfunded. Disease burdens (measured in disability associated life years, or DALYs) and international funding are not in synch: non-communicable diseases, with half the DALYs burden, currently get less than two percentage of global health funding.^36^

## CONCLUSIONS

Given the age pyramid in Nepal, a future-thinking approach to cancer in Nepal must focus on prevention. The role of both active and passive tobacco smoking in multiple major and common cancers needs to be part of public health messaging. More and stronger public health messaging about betel-nut chewing and home solid fuel burning is needed. Public policies must be developed to address also these two areas and HPV vaccination. The unfortunate circumstances of common stomach and gallbladder cancers should be the focus of collaborative international research activities.
